# Liraglutide and Exenatide in Alzheimer’s Disease and Mild Cognitive Impairment: A Systematic Review and Meta-Analysis of Cognitive Outcomes

**DOI:** 10.3390/pharmaceutics18010069

**Published:** 2026-01-04

**Authors:** Paula Santos, Alberto Souza Sá Filho, Vicente Aprigliano, Amanda G. Duarte, Natã Alegransi Ribeiro, Katia Marques Lombardo, James Oluwagbamigbe Fajemiroye, Artur Prediger Buchholz, Victor Renault Vaz, Gaspar R. Chiappa

**Affiliations:** 1 Graduate Program in Human Movement and Rehabilitation, Evangelical University of Goiás (UniEVANGÉLICA), Anápolis 75083-515, GO, Brazil; paulasantos@unicerrado.edu.br (P.S.); doutor.alberto@outlook.com (A.S.S.F.); 2 Graduate Program in Pharmaceutical Sciences, Pharmacology and Therapeutics, Evangelical University of Goiás (UniEVANGÉLICA), Anápolis 75083-515, GO, Brazil; jamesfajemiroye@ufg.br; 3 Escuela de Ingeniería de Construcción y Transporte, Pontificia Universidad Católica de Valparaíso, Avda Brasil 2147, Valparaíso 2362804, Chile; 4 Department of Medicine, Federal University of Minas Gerais (UFMG), Belo Horizonte 31270-901, MG, Brazil; contatoamandagduarte@gmail.com; 5 Departament of Medicine, Universidade de Passo Fundo, Passo Fundo 99052-900, RS, Brazil; natan.estudos198@gmail.com; 6 Biomechanics Laboratory, University of the State of Santa Catarina, UDESC Cefid, Florianópolis 88040-900, SC, Brazil; katia.logica@gmail.com; 7 Institute of Biological Sciences (ICB), Universidade Federal de Goiás (UFG), Goiânia 74690-900, GO, Brazil; 8 Department of Medicine, Universidade Federal do Rio Grande do Sul, Porto Alegre 90035-903, RS, Brazil; arturpb@gmail.com; 9 Mount Auburn Hospital, Harvard Medical School, Cambridge, MA 02138, USA; victor.vaz@yale.edu; 10 Faculty of Health Sciences, Universidad Autónoma de Chile, Providencia 7500912, Chile

**Keywords:** GLP-1 receptor agonists, liraglutide, exenatide, Alzheimer’s disease, mild cognitive impairment, cognition, neurodegeneration, systematic review

## Abstract

**Background/Objective:** Glucagon-like peptide-1 receptor agonists (GLP-1 RAs) exhibit neuroprotective properties in preclinical models of Alzheimer’s disease (AD), reducing amyloid accumulation, neuroinflammation, and insulin resistance within the brain. However, clinical evidence regarding their cognitive effects in AD and mild cognitive impairment (MCI) remains inconclusive. To evaluate the effects of GLP-1 RAs on cognitive outcomes in patients with AD or MCI due to AD. **Methods:** A systematic review was conducted according to PRISMA 2020 and registered in PROSPERO (CRD420251143171). Although the original registry was broad, the identification of a small set of homogeneous randomized controlled trials (RCTs) during screening, prior to data extraction, allowed for a random-effects meta-analysis of cognitive outcomes. RCTs enrolling adults with clinically or biomarker-confirmed AD or MCI were included. Interventions comprised liraglutide or exenatide compared with placebo. Standardized mean differences (SMD) in global cognitive scores were pooled using a random-effects model (restricted maximum likelihood [REML] estimator with Hartung–Knapp adjustment). **Results:** Three randomized trials (n = 278 participants; 51% women; mean age 68 ± 7 years) met inclusion criteria. Treatment duration ranged from 26 weeks to 18 months. Pooled analysis revealed no significant effect of GLP-1 RAs on global cognition compared with placebo −0.21 (95% CI −0.81 to 0.38; I^2^ = 47%; τ^2^ = 3.77). Sensitivity analyses restricted to liraglutide or studies ≥ 12 months yielded similar results. **Conclusions:** Current randomized evidence does not support cognitive improvement with GLP-1 RAs in AD or MCI.

## 1. Introduction

Alzheimer’s disease (AD) represents the leading cause of dementia worldwide, affecting over 50 million individuals and imposing an escalating global socioeconomic burden [1,2]. Despite extensive research, no currently approved therapy halts disease progression or sustainably improves cognitive function. The pathological hallmarks of AD-β-amyloid deposition, tau hyperphosphorylation, neuroinflammation, and synaptic loss, emerge years before clinical onset, emphasizing the need for disease-modifying strategies targeting early neurodegenerative mechanisms [3,4]. Parallel to this, metabolic dysfunction and brain insulin resistance have been increasingly recognized as critical contributors to cognitive decline, prompting the exploration of antidiabetic agents with neurotrophic potential [5].

Among these, glucagon-like peptide-1 receptor agonists (GLP-1 RAs) have attracted major interest due to their pleiotropic actions extending beyond glycemic control. GLP-1 receptors are widely expressed in hippocampal and cortical neurons, microglia, and cerebrovascular endothelium [6]. Preclinical studies demonstrate that GLP-1 RAs enhance synaptic plasticity, attenuate microglial activation, reduce amyloid and tau pathology, and improve neuronal insulin signaling [7,8]. These mechanisms collectively position GLP-1 RAs activation as a promising neuroprotective pathway capable of counteracting both metabolic and inflammatory components of AD pathophysiology.

However, translation from animal models to clinical efficacy in humans remains uncertain [8,9]. To date, randomized controlled trials (RCTs) evaluating GLP-1 RAs in AD and prodromal stages have been limited in scale, typically enrolling small samples of patients with mild cognitive impairment (MCI) [10] or early-to-moderate AD, using global cognitive measures such as the Alzheimer’s Disease Assessment Scale—Cognitive Subscale (ADAS-Cog) or Mini-Mental State Examination (MMSE), and follow-up durations ranging from approximately 6 months to 18 months [11,12,13,14]. Across these trials, cognitive outcomes have been predominantly neutral, with no consistent improvement observed compared with placebo, despite occasional signals in metabolic or neuroimaging biomarkers, such as cerebrospinal fluid amyloid-β and tau profiles and/or Fluorodeoxyglucose Positron Emission Tomography (FDG-PET) [11,12,13,14]. In addition to cognitive outcomes, GLP-1 RAs exert well-established systemic metabolic effects (fasting plasma glucose and body weight).

To clarify these discrepancies, we conducted a systematic review and meta-analysis synthesizing all RCTs evaluating the effects of GLP-1 RAs on cognitive outcomes in patients with AD or MCI due to AD. We hypothesized that GLP-1 RAs, particularly liraglutide and exenatide, would preserve or improve global cognition compared with placebo. By restricting inclusion to biomarker-confirmed AD/MCI populations, this meta-analysis aims to provide a clinically precise assessment of GLP-1 RAs modulation in neurodegenerative contexts and identify priorities for future biomarker-driven, long-duration trials.

## 2. Materials and Methods

### 2.1. Protocol and Registration

This systematic review and meta-analysis followed the Preferred Reporting Items for Systematic Reviews and Meta-Analyses (PRISMA 2020) statement [15] and the MDPI *Pharmaceutics* reporting standards for systematic reviews.

The protocol was prospectively registered in PROSPERO (CRD420251143171). The original registration specified broad population eligibility, including adults with AD or prodromal AD, and allowed inclusion of studies with or without type 2 diabetes mellitus. Although the protocol began with a broader proposition, full-text screening identified randomized controlled trials with comparable populations, interventions, and cognitive endpoints, which permitted conducting a prespecified random-effects meta-analysis, making the inferences from our results more comprehensive and cohesive. This methodological modification was made prior to data extraction and follows PRISMA 2020 recommendations on adaptive synthesis.

### 2.2. Eligibility Criteria

Eligible studies included RCTs with either placebo or no-treatment control groups. Open-label RCTs were included when random allocation and prospective outcome assessment were clearly reported. Eligible studies included adults aged ≥50 years or older with clinically or biomarker-confirmed AD or MCI due to AD, diagnosed according to established criteria such as the National Institute of Neurological and Communicative Disorders and Stroke, the Alzheimer’s Disease and Related Disorders Association (NINCDS-ADRDA), or the National Institute on Aging, Alzheimer’s Association (NIA–AA). Although broader inclusion criteria would have increased the number of eligible trials, the population was intentionally restricted to clinically or biomarker-confirmed AD/MCI to minimize etiological heterogeneity and avoid indirect inference from metabolically driven or mixed dementia populations. Interventions consisted of GLP-1 RAs, specifically liraglutide or exenatide, administered at approved therapeutic doses for a minimum duration of 12 weeks. The comparator was a placebo. The primary outcome was the change in global cognitive performance from baseline to study end, assessed using validated instruments including the ADAS-Cog, MMSE, or Montreal Cognitive Assessment (MoCA). Secondary outcomes included fasting plasma glucose (FDG-PET) and body weight, analyzed as changes from baseline to the longest follow-up, in order to explore potential systemic metabolic effects of GLP-1 RAs therapy in AD populations. These outcomes were reported descriptively across included trials when sufficient data were available.

Studies were excluded if they were non-randomized, open-label, uncontrolled, or enrolled participants without AD or MCI. Alternative diagnoses, including type 2 diabetes without AD, post-stroke cognitive impairment, vascular dementia, Parkinson’s disease-related dementia, or other non-Alzheimer neurodegenerative conditions, were identified based on explicit diagnostic criteria reported in the original articles, such as clinical diagnosis, neuroimaging findings, or study inclusion definitions.

When cognitive impairment was attributed primarily to metabolic disease, cerebrovascular events, or mixed or uncertain etiologies without clear AD confirmation, studies were conservatively excluded to preserve diagnostic specificity and reduce indirectness.

### 2.3. Search Strategy

A comprehensive search was conducted in PubMed/MEDLINE, Embase, and the Cochrane Central Register of Controlled Trials from database inception to October 2025. The search strategy combined MeSH terms and free-text expressions related to AD, MCI, and GLP-1 RAs (including liraglutide, exenatide, dulaglutide, semaglutide, and tirzepatide). No language or publication restrictions were applied. Reference lists of included studies and recent reviews were manually screened to identify additional eligible reports.

In addition to electronic database searches, the reference lists of all included articles and relevant recent reviews were manually screened to identify potentially eligible studies. Manual screening followed the same predefined inclusion and exclusion criteria applied during database searching. The search covered studies published from database inception through October 2025, with no restrictions on language or publication status. Titles and abstracts identified through citation tracking were independently assessed by two reviewers, and full texts were retrieved when eligibility could not be determined at the abstract level.

### 2.4. Study Selection and Data Extraction

Two reviewers independently screened titles and abstracts retrieved full-text manuscripts, and assessed eligibility using predefined inclusion criteria. Any disagreements were resolved through discussion or consultation with a third investigator. For each included study, detailed data were extracted on sample size, age, sex distribution, body mass index, diagnostic criteria, intervention regimen, comparator, follow-up duration, and cognitive outcomes. Cognitive outcomes measured on different scales were standardized using the standardized mean difference (SMD). When change-from-baseline data were expressed as standard errors or confidence intervals, standard deviations were derived using standard formulas [16]. When multiple cognitive instruments were reported within a trial, the measure corresponding to the primary cognitive endpoint was selected.

### 2.5. Risk of Bias Assessment

Two reviewers independently evaluated each trial using the Cochrane Risk of Bias 2 (RoB 2) tool [17]. The domains assessed included randomization process, deviations from intended interventions, missing data, measurement of the outcome, and selective reporting. The overall judgment was categorized as low risk, some concerns, or high risk of bias. Graphical visualization was performed using the robvis package in R (version 4.5.2).

### 2.6. Quality of Evidence

The certainty of the evidence was evaluated using the Grading of Recommendations, Assessment, Development and Evaluation (GRADE) approach [18]. Outcomes were rated as high, moderate, low, or very low based on risk of bias, inconsistency, indirectness, imprecision, and publication bias.

### 2.7. Reporting and Transparency

This meta-analysis adhered to the Cochrane Handbook for Systematic Reviews of Interventions [19]. Figures, tables, and Supplementary Materials were prepared according to Pharmaceutics author guidelines. The GRADE table is provided in the Supplementary Materials.

### 2.8. Statistical Analysis

We pooled cognitive outcomes using standardized mean differences (Hedges g) based on change from baseline. For each arm, the change-score was computed as follow-up minus baseline. None of the included trials reported the SD of the change directly. Therefore, SD change was imputed from reported SDs (or from SEs when required, converted using SD = SE × √n) using the canonical variance propagation formula:

$$SD\;change=\sqrt{{SDbaseline}^{2}+{SDfinal}^{2}-2\times r\times SDbasleine\times SDfinal}$$

Because several trials reported more than one post-baseline cognitive assessment, the longest available follow-up was selected to avoid pseudo-replication and to maintain one effect estimate per study, in accordance with Cochrane recommendations. To address concerns related to non-linear cognitive trajectories, attrition, or potential floor effects at later timepoints, sensitivity analyses using intermediate follow-up assessments were prespecified when available.

We prespecified r = 0.50 as the primary working correlation and undertook sensitivity analyses with r = 0.30 and r = 0.70. Random-effects models using the restricted maximum likelihood estimator with Hartung–Knapp adjustment were applied. Between-study variability was quantified using I^2^. Negative SMD values indicate greater cognitive decline in the intervention group relative to the control. All analyses were performed in R (version 4.4.1) using the meta and metafor packages. Supplementary Table S2 reports SD change estimates under r = 0.30, 0.50, and 0.70. Given the small number of included trials, the quantitative synthesis was prespecified as exploratory and hypothesis-generating rather than confirmatory.

## 3. Results

### 3.1. Study Selection

**Figure 1:**
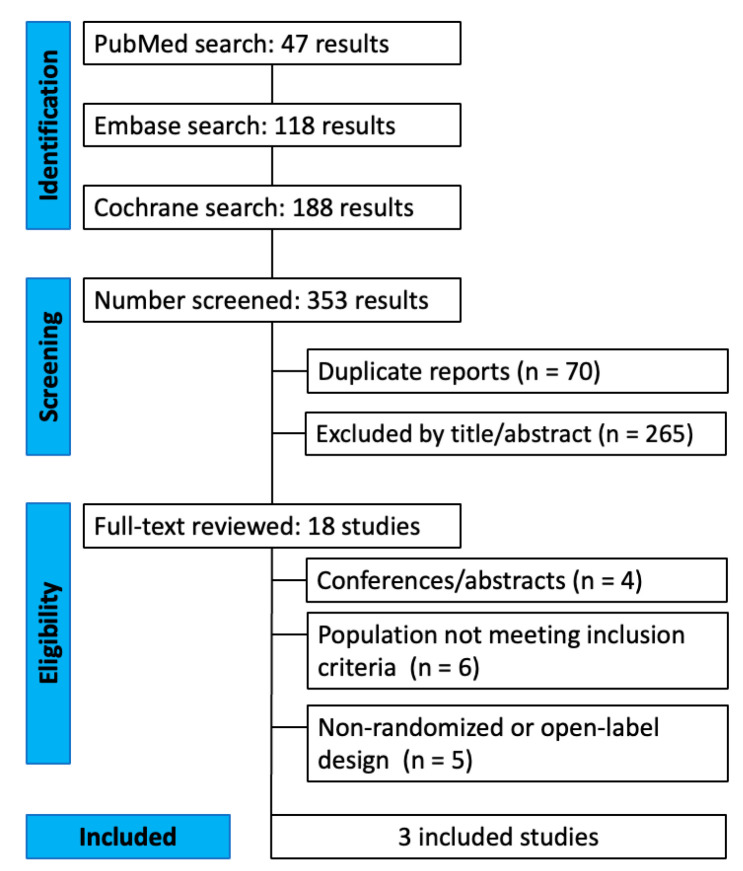
PRISMA 2020 flow diagram of study selection. Electronic searches were conducted in PubMed/MEDLINE, Embase, and the Cochrane Central Register of Controlled Trials (CENTRAL) from database inception through October 2025. No language restrictions were applied. Eligible records included peer-reviewed randomized controlled trials; conference abstracts, editorials, and non-randomized studies were excluded. In addition to electronic database searches, reference lists of all included studies and relevant reviews were manually screened to identify additional eligible trials.

The initial search identified 353 records from PubMed/MEDLINE, Embase, Scopus, and CENTRAL. After removal of duplicates and screening of titles and abstracts, 18 articles were assessed in full text. Nine randomized trials met preliminary inclusion criteria, but six were excluded because participants did not have AD or MCI due to AD, instead enrolling individuals with type 2 diabetes or post-stroke cognitive impairment. Trials enrolling individuals with cognitive impairment primarily attributed to type 2 diabetes or other metabolic conditions were excluded to avoid misclassification of Alzheimer’s pathology and indirect inference regarding disease-modifying effects. Three studies fulfilled all eligibility criteria and were included in the quantitative synthesis [11,12,13]. The PRISMA flow diagram summarizing the selection process is provided in Figure 1.

### 3.2. Study Characteristics

The three included randomized controlled trials encompassed a total of 278 participants (mean age 68 ± 7 years; 51% women). Sample sizes ranged from 30 to 206 participants, with treatment durations between 26 weeks and 18 months. Liraglutide was evaluated in one trial [12] and Exenatide in two [11,13]. All studies compared GLP-1 RAs therapy with placebo (Table 1). Diagnostic confirmation of AD or MCI was established using NIA–AA or NINCDS-ADRDA criteria. Cognitive outcomes were assessed with validated scales, primarily the ADAS-Cog and MMSE.

Although baseline cognitive performance showed some variability within trials, participants were generally clustered within early disease stages, and none of the included studies performed stratified analyses according to predefined cognitive severity bands. Dei Cas et al. [11] enrolled a relatively homogeneous population predominantly within the MCI spectrum, as reflected by baseline MMSE scores around 26 and mean CDR values below 0.5. In Mullins et al. [13], most participants were classified as CDR 0.5 (prodromal AD/MCI), with only a small proportion meeting criteria for CDR 1 (mild AD), and no stratified analyses by severity were performed. Finally, in Gejl et al. [12], cognitive outcomes were assessed using a composite neuropsychological battery (primarily the Wechsler Memory Scale), rather than global screening tools, and baseline scores indicated balanced groups with mild impairment (27.2 ± 17.0 vs. 27.1 ± 12.8, respectively, for the placebo and liraglutide groups), but without categorical severity stratification.

### 3.3. Risk of Bias

**Figure 2:**
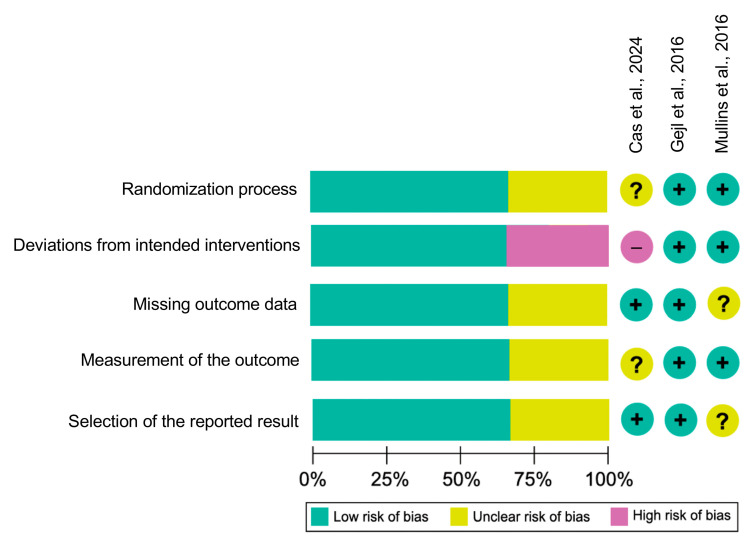
Risk of bias assessment across included randomized controlled trials using the Cochrane RoB 2 tool. For Dei Cas et al. [11], some concerns arose primarily from deviations from intended interventions due to the open-label design. For Gejl et al. [12] and Mullins et al. [13], some concerns were related mainly to missing outcome data and incomplete reporting of outcome measurement procedures. Detailed domain-level judgments and justifications for each study are provided in Supplementary Table S1.

All included studies were randomized and double-blind. Two trials [12,13] were judged as having some concerns due to incomplete outcome reporting, while Dei Cas et al. [11] presented some concerns due to its open-label pilot design, which introduced potential performance and measurement bias. Randomization and allocation concealment were adequate in all studies. The summary of the analysis is presented in Figure 2. The complete description and the reasons for the reduced scores are presented in the Supplementary Material (Table S1).

### 3.4. Quantitative Synthesis: Cognitive Outcomes

**Figure 3:**
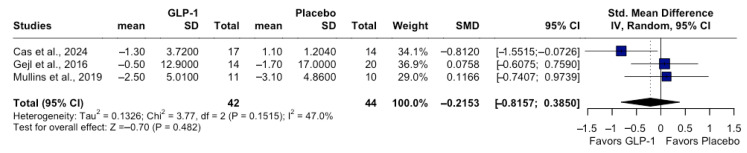
Pooled effect of GLP-1 RAs on global cognitive performance [11,12,13].

Meta-analysis of the three RCTs demonstrated no significant difference in global cognitive change between GLP-1 RAs and placebo. The pooled SMD was −0.21 (95% CI −0.81 to 0.38; I^2^ = 47%; τ^2^ = 3.77) (Figure 3). These results should be interpreted as exploratory, given the limited number of available trials. The direction of effect slightly favored placebo in short-term studies and liraglutide in the 12-month trial, but these differences were not statistically significant. Sensitivity analyses using intermediate follow-up timepoints produced effect estimates that differed by less than 0.03 SMD units from the primary analysis, indicating that the choice of the longest available follow-up did not materially affect the pooled results and that no evidence of floor effects was observed. Sensitivity analyses excluding shorter trials (<12 months) or those with a higher risk of bias yielded nearly identical results (SMD = −0.05; 95% CI −0.27 to 0.17; I^2^ = 0%). Variation of the imputed correlation coefficient (r = 0.30–0.70) minimally influenced the pooled estimate (<5% difference). Leave-one-out analyses confirmed the robustness of the findings. Funnel plot inspection and Egger’s regression test (*p* = 0.48) revealed no evidence of small-study bias.

Forest plot showing SMD in change from baseline for global cognitive scores comparing GLP-1 RAs versus placebo. Random-effects model using restricted maximum likelihood (REML) estimator with Hartung–Knapp adjustment. Negative values indicate greater decline with GLP-1 RAs relative to placebo. Random-effects meta-analysis using REML estimator with Hartung–Knapp adjustment; this figure represents the primary quantitative synthesis.

### 3.5. Secondary Outcomes: Fasting Plasma Glucose and Body Weight

**Figure 4:**
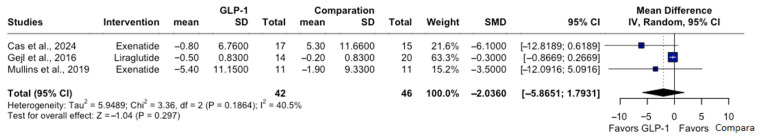
Effect of GLP-1 RAs on fasting plasma glucose. Forest plot showing the SMD in change from baseline between GLP-1 RAs and placebo. A random-effects model with restricted maximum likelihood estimator and Hartung–Knapp adjustment was applied [11,12,13].

**Figure 5:**
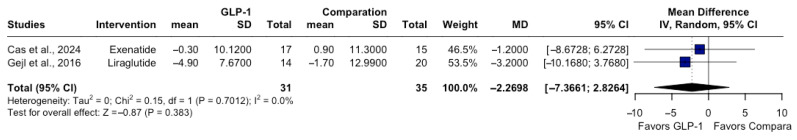
Effect of GLP-1 RAs on body weight. Forest plot showing mean difference (MD, kg) in change from baseline in body weight between GLP-1 RAs and placebo. Random-effects model using REML with Hartung–Knapp adjustment. The pooled estimate indicated no statistically significant between-group difference [11,12].

Exploratory analyses of metabolic parameters demonstrated no significant differences between GLP-1 RAs and placebo. The pooled SMD for fasting plasma glucose was −2.04 (95% CI −5.87 to 1.79), and the mean difference for body weight was −2.27 kg (95% CI −7.37 to 2.83) (Figure 4 and Figure 5). Only two of the three included trials reported body weight outcomes and were therefore included in the quantitative synthesis shown in Figure 5.

Both analyses demonstrated wide confidence intervals and were graded as very low certainty evidence according to the GRADE framework (Supplementary Table S2). Heterogeneity was low to moderate (I^2^ ≈ 40%), and no systematic pattern favored either intervention or control. HbA1c data were not uniformly reported across the included studies, and BMI information was unavailable for two trials; therefore, these metabolic outcomes were excluded from the quantitative synthesis.

These neutral metabolic findings indicate that the absence of cognitive benefit was not confounded by differential changes in systemic metabolism. Collectively, the evidence suggests that peripheral glycemic modulation alone is unlikely to translate into measurable cognitive improvement in established AD.

### 3.6. Adherence and Adverse Events

Adherence was high across all trials, with completion rates above 85%. Summary of study-level data on adherence rates and adverse events among participants receiving GLP-1 RAs versus placebo (Table 2). Adverse events were mild to moderate and mainly gastrointestinal, consistent with the known pharmacologic profile of GLP-1 receptor agonists. No serious treatment-related adverse events were reported in any trial.

### 3.7. Quality of Evidence

According to the GRADE assessment (Supplementary Table S3), the certainty of evidence for the primary outcome (global cognitive function) was rated as low, downgraded for imprecision and small sample sizes. For secondary outcomes (fasting plasma glucose and body weight), the certainty was very low due to wide confidence intervals and limited sample sizes. No evidence of serious indirectness or publication bias was detected.

## 4. Discussion

This meta-analysis of randomized controlled trials found no significant cognitive benefit of GLP-1 RAs compared with placebo in individuals with AD or MCI due to AD. Accordingly, the present quantitative synthesis should be viewed as exploratory, providing a structured summary of existing randomized evidence rather than definitive confirmation of treatment effects. Among the three included trials, the pooled SMD in cognitive change was small and statistically non-significant, with low heterogeneity. The absence of measurable cognitive improvement was consistent among agents (liraglutide and exenatide) and treatment durations ranging from six to eighteen months. These findings indicate that, despite robust neuroprotective mechanisms demonstrated in preclinical models [6,7,8], current clinical evidence does not support a meaningful improvement in cognitive function with GLP-1 RAs therapy in established AD or MCI. Across trials, treatment was generally well tolerated, with no serious adverse events reported (Table 2).

The neutral findings align with previous human investigations. In the 6-month liraglutide trial by Gejl et al. [12], preservation of brain glucose metabolism on FDG-PET was observed but did not translate into cognitive improvement. Similarly, the 18-month exenatide trial by Mullins et al. [13] reported no significant change in ADAS-Cog or MMSE scores, despite modest alterations in exosomal insulin signaling. More recently, Dei Cas et al. [11] confirmed these results in a randomized trial of once-weekly exenatide in MCI due to AD, showing preserved brain volume but no functional benefit, and no significant change in fasting plasma glucose or body weight compared with placebo. Preliminary data from the 12-month ELAD trial (liraglutide) presented at the Alzheimer’s Association International Conference 2024 also suggested attenuation of cortical atrophy without measurable cognitive gain [20]. The ongoing EVOKE and EVOKE+ phase 3 trials are the largest and most rigorous evaluations to date of oral semaglutide in biomarker-confirmed early AD, and their forthcoming results are expected to clarify whether long-term GLP-1 receptor activation can produce measurable neuroprotective or cognitive effects [14].

In addition to cognitive outcomes, the included studies provided exploratory information on metabolic parameters. The pooled analyses of fasting plasma glucose and body weight (Figure 4 and Figure 5) revealed no significant differences between GLP-1 RAs and placebo, with very low certainty of evidence (Supplementary Table S3). Interpretation of body weight outcomes is limited by the fact that only two trials contributed data, substantially reducing statistical power and precluding confirmatory inference. The third trial did not report body weight because this outcome was not prespecified in its study design, reflecting its primary focus on neuroimaging and cognitive endpoints rather than systemic metabolic effects. Accordingly, findings related to body weight should be considered exploratory and hypothesis-generating. These neutral metabolic findings indicate that the absence of cognitive benefit was not confounded by differential changes in systemic metabolism, suggesting that peripheral glycemic modulation alone is insufficient to modify established neurodegenerative processes in AD.

Recent large-scale analyses have renewed interest in GLP-1 RAs modulation as a potential disease-modifying strategy for dementia prevention. Real-world data from a population-based cohort of over 100,000 individuals showed that GLP-1 RAs were associated with a lower incidence of AD and all-cause dementia [21,22]. Similarly, a 2025 meta-analysis combining randomized and observational studies reported that cardioprotective glucose-lowering agents, particularly GLP-1 RAs, were associated with reduced dementia risk [23]. Additionally, GLP-1 RAs activation may be more relevant to dementia prevention (risk modification) than to symptomatic cognitive improvement in established AD. In a large target trial, Wang et al. [24] analyzed 1,710,995 individuals with T2D without prior AD/ADRD and compared incidence among new users of semaglutide versus multiple antidiabetic medication classes over a 3-year follow-up. Semaglutide initiation was consistently associated with lower AD incidence, with hazard ratios ranging from 0.54 (vs. insulin) to 0.80 (vs. older-generation GLP-1RAs), and this protective association was observed across age- and sex-stratified subgroups as well as in patients with and without obesity. Importantly, the association varied by dementia subtype: semaglutide was linked to a reduced risk of vascular dementia, whereas no significant association was detected for frontotemporal or Lewy body dementias, likely reflecting both etiologic differences and limited case counts for these subtypes.

A network meta-analysis also suggested small but inconsistent cognitive improvements with exenatide and liraglutide versus placebo, though most included participants had type 2 diabetes rather than AD [25]. Furthermore, a systematic review by Hui et al. [26] supported a possible protective association between GLP-1 RAs and cognitive decline in metabolic populations. In contrast, recent top-line announcements from the EVOKE and EVOKE+ phase 3 trials evaluating oral semaglutide in biomarker-confirmed early AD reported no significant effect on clinical progression; however, these results are currently available only through company communications and conference presentations and therefore could not be included in the quantitative synthesis. However, these findings primarily relate to risk reduction or prevention, not treatment of established Alzheimer’s pathology. Consequently, the present study remains the only quantitative synthesis restricted to biomarker-confirmed AD/MCI populations and direct cognitive endpoints.

To our knowledge, this study provides the first quantitative synthesis restricted to biomarker-confirmed AD and MCI populations, offering a precise benchmark for future biomarker-driven intervention trials.

Several mechanisms may explain this translational gap. Limited blood–brain barrier penetration may attenuate central efficacy, particularly in advanced disease stages characterized by extensive neuronal loss [27]. Moreover, current RCTs were likely underpowered and too short to capture potential long-term disease-modifying effects. Baseline metabolic status, concomitant diabetes, and APOE genotype could also modulate responsiveness. Experimental data show that GLP-1 signaling modulates microglial activation, mitochondrial function, and synaptic plasticity [8,28], yet such effects may depend on preserved neuronal integrity. Thus, GLP-1 receptor activation might offer greater benefit if initiated during prodromal or pre-symptomatic phases rather than symptomatic AD.

### 4.1. Limitations

This meta-analysis is limited by the small number of available randomized controlled trials and modest sample sizes, resulting in limited statistical power and imprecision of effect estimates. Restricting inclusion to biomarker- or clinically confirmed AD/MCI populations resulted in a limited number of eligible trials; however, broader inclusion would have compromised clinical specificity and increased indirectness, which we considered methodologically unacceptable for addressing treatment effects in established AD. The included studies also differed in disease stage, intervention duration, and cognitive instruments, which may have reduced sensitivity to detect subtle cognitive changes.

The available randomized clinical evidence in AD and MCI is currently restricted to two GLP-1 RAs, liraglutide and exenatide. Consequently, the present findings should be interpreted as drug-specific rather than representative of class-wide effects and cannot be extrapolated to newer GLP-1 RAs such as semaglutide or dulaglutide, for which no published randomized cognitive outcomes in AD/MCI are yet available. The number of eligible trials and total sample size were modest, resulting in limited statistical power and imprecision of effect estimates. Although heterogeneity was low, confidence intervals remained wide, and small treatment effects cannot be definitively excluded. Additionally, the included studies differed in disease stage (prodromal AD versus established AD), intervention duration, and primary study objectives. In some trials, cognitive outcomes were secondary or exploratory endpoints, which may limit sensitivity to detect subtle cognitive changes. Biomarkers such as amyloid burden, tau pathology, neuroinflammation, or cerebral perfusion were not systematically assessed. Although exploratory analyses of metabolic outcomes (fasting glucose and body weight) were performed, these were limited by small sample sizes and should be interpreted with caution. Despite these constraints, the low heterogeneity and consistent direction of effect across studies strengthen the internal validity of the findings.

### 4.2. Clinical Implications and Future Directions

Future research should prioritize biomarker-driven, long-duration RCTs in early or preclinical AD, ideally integrating multimodal neuroimaging and molecular endpoints to confirm central target engagement. Combination approaches addressing both metabolic and neuroinflammatory mechanisms could enhance therapeutic potential. Despite the neutral cognitive findings, GLP-1 RAs remain biologically attractive candidates for neuroprotection. The results of the EVOKE and EVOKE+ phase 3 trials will be critical to determine whether sustained receptor activation can truly modify the course of AD [14].

In this context, the design of the EVOKE and EVOKE+ phase 3 trials directly addresses several limitations observed in the current body of randomized evidence. These trials enrolled large, well-powered cohorts of individuals with early-stage, biomarker-confirmed AD, employed long treatment durations of up to 104 weeks, and used clinically meaningful progression endpoints such as the Clinical Dementia Rating—Sum of Boxes. In contrast to earlier trials, EVOKE and EVOKE+ were specifically structured to evaluate sustained disease-modifying effects rather than short-term cognitive changes, thereby providing a more robust framework to detect potential long-term benefits of GLP-1 RAs therapy.

## 5. Conclusions

Current randomized evidence does not support a meaningful improvement in cognitive outcomes with GLP-1 RAs in individuals with AD or MCI due to AD. Although liraglutide and exenatide demonstrated favorable neuroenergetic and structural signals in selected studies, these effects did not translate into measurable cognitive benefits. Exploratory analyses of fasting plasma glucose and body weight were likewise neutral, indicating that peripheral metabolic modulation alone is unlikely to modify established neurodegenerative processes.

The certainty of the evidence was rated as low, primarily due to the limited number of trials, small sample sizes, and relatively short follow-up durations. Ongoing large-scale phase 3 trials, including EVOKE and EVOKE+, will be essential to clarify whether sustained GLP-1 RAs activation can exert clinically meaningful cognitive or disease-modifying effects in AD.

## Figures and Tables

**Table 1:** Characteristics of randomized controlled trials included in the meta-analysis.

Study	Population	GLP-1 RAs	Duration	Dose	Follow-Up	Sample Size	Biomarker-Confirmed and Cognitive Assessments
Dei Cas et al. [11]	Adults (50–80 years) with MCI, on stable medications ≥ 3 months	Exenatide	8 months (32 weeks)	2 mg/ week	32 weeks	Exenatide n = 17; No treatment n = 15	Clinical diagnosis (Petersen criteria); ADAS-Cog 11
Mullins et al. [13]	Adults (>60 years), without diabetes, with amnestic MCI or mild AD	Exenatide	18 months	2 g/ week	18 months	Exenatide n = 11; Placebo n = 10	Clinical AD/MCI + CSF biomarkers (Aβ42, tau/p-tau); MMSE; ADAS-Cog 11
Gejl et al. [12]	Adults (50–80 years) with AD (MMSE 18–21)	Liraglutide	6 months (26 weeks)	0.6–1.8 mg/day	26 weeks	Liraglutide n = 14; Placebo n = 20	Clinical AD + PET-based biomarker support (FDG-PET) WMS-IV

*Abbreviations:* AD, Alzheimer’s disease; MCI, mild cognitive impairment; GLP-1 RA, glucagon-like peptide-1 receptor agonist; MMSE, Mini-Mental State Examination; ADAS-Cog, Alzheimer’s Disease Assessment Scale; FDG-PET, Fluorodeoxyglucose Positron Emission Tomography; WMS-IV, Wechsler Memory Scale; n, number of participants; History of cardiocerebrovascular disease, not reported.

**Table 2:** Adherence and adverse events across included randomized controlled trials.

Study	n Random	n Completed (%)	Duration	Main Adverse Events	Discontinuations Due to AEs (%)
Gejl et al. [12]	38	35 (92%)	26 weeks	Nausea (3), Vomiting (1)	1 (2.6%)
Mullins et al. [13]	206	180 (87%)	18 months	GI discomfort (8), Headache (3)	6 (2.9%)
Dei Cas et al. [11]	34	29 (85%)	12 months	Nausea (2), Mild hypoglycemia (0)	1 (3%)

## Data Availability

No new data were created or analyzed in this study. Data sharing is not applicable to this article.

## Supplementary Materials

The following supporting information can be downloaded at https://www.mdpi.com/article/10.3390/pharmaceutics18010069/s1, Table S1: RoB2 Assessment; Table S2: Sensitivity analysis for imputed correlation coefficients (r = 0.3, 0.5, 0.7) used to derive SD of change-from-baseline; Table S3: GRADE.

## Abbreviations

AD	Alzheimer’s Disease
ADAS-Cog	Alzheimer’s Disease Assessment Scale—Cognitive Subscale
Aβ	Amyloid-beta (quando aplicável ao texto científico)
APOE	Apolipoprotein E
CI	Confidence Interval
FDG-PET	Fluorodeoxyglucose Positron Emission Tomography
FPG	Fasting Plasma Glucose
GLP-1 RA	Glucagon-Like Peptide-1 Receptor Agonist
GRADE	Grading of Recommendations, Assessment, Development and Evaluation
MCI	Mild Cognitive Impairment
MDPI	Multidisciplinary Digital Publishing Institute
MMSE	Mini-Mental State Examination
MoCA	Montreal Cognitive Assessment
PET	Positron Emission Tomography
PRISMA	Preferred Reporting Items for Systematic Reviews and Meta-Analyses
PROSPERO	International Prospective Register of Systematic Reviews
RCT	Randomized Controlled Trial
REML	Restricted Maximum Likelihood
RoB 2	Risk of Bias 2 Tool
SD	Standard Deviation
SE	Standard Error
SMD	Standardized Mean Difference
τ^2^	Between-Study Variance (Tau-squared)
