# Supplementary material for: Liraglutide and Exenatide in Alzheimer’s Disease and Mild Cognitive Impairment: A Systematic Review and Meta-Analysis of Cognitive Outcomes
Source: Pharmaceutics. 2026 Jan 4;18(1):69. doi: 10.3390/pharmaceutics18010069 (PMC12844791; doi:10.3390/pharmaceutics18010069)
Supplement: Supplementary file 1 [file pharmaceutics-18-00069-s001.zip › pharmaceutics-4021808-supplementary.pdf]

SUPPLEMENTARY MATERIAL

Supplementary File (PDF)

Supplementary Table S1. RoB2 Assessment

Supplementary Table S2. Sensitivity analysis for imputed correlation coefficients

Supplementary Table S3. GRADE summary of findings

Table S1. RoB2 Assessment

Domain	Cas et al., 2024	Gejl et al., 2016	Mullins et al., 2019
**1. Randomization process**	**Some concerns** – randomization method not fully described; unclear allocation concealment.	**Low risk** – described random sequence and concealment in protocol.	**Low risk** – reported random sequence generation and allocation concealment.
**2. Deviations from intended interventions**	**High risk** – "no treatment" control arm, open-label; participants and personnel aware of group allocation.	**Low risk** – double-blind design with matched placebo.	**Low risk** – double-blind and placebo-controlled.
**3. Missing outcome data**	**Low risk** – dropouts <10%, reasons balanced between groups.	**Low risk** – low attrition; intention-to-treat applied.	**Some concerns** – 2 dropouts in GLP-1 arm (≈15%) without clear imputation procedure.
**4. Measurement of the outcome**	**Some concerns** – cognitive testing not blinded; potential assessor bias possible.	**Low risk** – blinded outcome assessors for cognitive and metabolic measures.	**Low risk** – assessor blinding reported for all endpoints.
**5. Selection of the reported result**	**Low risk** – protocol publicly available, prespecified outcomes.	**Low risk** – consistent with published protocol (ClinicalTrials.gov NCT01469351).	**Some concerns** – minor deviations in reported timepoints (6, 12, 18 months).
**Overall risk of bias (RoB2)**	**High** – due to open-label design and potential measurement bias.	**Low** – fully blinded, consistent reporting.	**Some concerns** – attrition and selective timing in reporting.

Table S2. Sensitivity analysis for imputed correlation coefficients (r = 0.3, 0.5, 0.7) used to derive SD of change-from-baseline

Outcome	Study	Group	r = 0.3 SDΔ	r = 0.5 SDΔ	r = 0.7 SDΔ	Direction of effect	Impact on pooled estimate
**Cognition (SMD)**	Gejl 2016	Liraglutide vs placebo	4.55	3.70	2.60	↓ favors GLP-1	Minimal – SMD shifts <0.05
	Cas 2024	Exenatide vs no treatment	6.10	4.90	3.45	↓ favors GLP-1	Minimal
	Mullins 2019	Exenatide vs placebo	7.20	5.80	4.10	↓ favors GLP-1	Minimal
**FPG (SMD)**	Gejl 2016	Liraglutide vs placebo	8.50	6.90	4.90	↓ favors GLP-1	<5% change in pooled SMD
	Cas 2024	Exenatide vs no treatment	9.10	7.40	5.30	↓ favors GLP-1	<10% change
	Mullins 2019	Exenatide vs placebo	11.0	8.90	6.20	↓ favors GLP-1	<10% change
**Weight (MD, kg)**	Gejl 2016	Liraglutide vs placebo	5.20	4.20	3.00	↓ favors GLP-1	negligible (<2%)
	Cas 2024	Exenatide vs no treatment	7.60	6.20	4.40	↓ favors GLP-1	negligible (<2%)

Table S3. GRADE

Review question: *Do GLP-1 receptor agonists improve cognition in individuals with mild cognitive impairment in early Alzheimer's disease?*

Summary of Findings Table (SoF)

Outcome (longest follow-up)	Participants (studies)	Risk of bias	Inconsistency	Indirectness	Imprecision	Publication bias	Effect (random-effects model)	Certainty (GRADE)
**Cognitive function (SMD, change from baseline)**	86 (3)	Serious[1]	Serious[2]	Not serious[3]	Serious[4]	Not assessable[5]	SMD −0.22 [−0.82; 0.39]	Low
**Fasting plasma glucose (SMD, change from baseline)**	88 (3)	Serious[1]	Possible[6]	Very serious[7]	Very serious[7]	Not assessable[5]	SMD −2.04 [−5.87; 1.79]	Very low
**Body weight (MD, kg, change from baseline)**	66 (2)	Serious[1]	Not serious[8]	Not serious	Serious[9]	Not assessable[5]	MD −2.27 [−7.37; 2.83]	Very low

Explanations and Justifications

1. **Risk of bias (−1):** Small sample sizes; one trial with a no-treatment control (Cas 2024) and unclear blinding. Change-score SDs were imputed.
2. **Inconsistency (−1):** Moderate heterogeneity ($I^2 \approx 47\%$) across cognitive outcomes using different instruments (MMSE vs WMS-IV).
3. **Indirectness:** All studies assessed validated cognitive or metabolic endpoints directly relevant to the target population.
4. **Imprecision (−1):** Wide 95% CIs crossing the null; total N insufficient to detect modest effects.
5. **Publication bias:** Fewer than 10 studies; funnel plots not interpretable. Not downgraded but labeled "not assessable."
6. **Inconsistency (possible):** Moderate heterogeneity ($I^2 \approx 40\%$) with variability in variance magnitude; retained as "possible."
7. **Imprecision (−2):** Extremely wide confidence interval (−5.9 to +1.8 SMD) compatible with both large benefit and harm; very low N.
8. **Inconsistency (not serious):** $I^2 \approx 0\%$; consistent direction of effect.
9. **Imprecision (−1):** Wide CI (−7.4 to +2.8 kg) crossing the null; total N small.
